# Sperm-Borne Mitochondrial Activity Influenced by Season and Age of Holstein Bulls

**DOI:** 10.3390/ijms252313064

**Published:** 2024-12-05

**Authors:** Khurshaid Anwar, Georg Thaller, Mohammed Saeed-Zidane

**Affiliations:** Molecular Genetics Group, Institute of Animal Breeding and Husbandry, Christian-Albrechts-University Kiel, 24118 Kiel, Germany

**Keywords:** male fertility, environmental factors, sperm-mitochondrial DNA, protein-coding gene, pathway enrichment

## Abstract

Sperm mitochondria are vital organelles for energy production and pre- and post-fertilization sperm functions. The potential influence of the age of the bull and season on the sperm-borne mitochondrial copy number and the transcription activity has not yet been investigated. Therefore, the expression patterns of all protein-coding mitochondrial genes were identified throughout the year along with mitochondrial copy numbers in young and old bulls’ spermatozoa. For that, high-quality semen samples (*n* = 32) with more than 80% quality for the morphological parameters, from young (*n* = 4, aged 18–24 months old) and old (*n* = 4, aged 40–54 months old) Holstein bulls, were collected during the four seasons (*n* = 4 samples each animal/season). The DNA and RNA were isolated from sperm cells and subjected to the DNA copy number and expression analyses using qPCR. Furthermore, an in silico analysis using gene ontology online tools for the abundantly expressed genes was utilized. The data were statistically analyzed using Prism10 software. There was a significant reduction in the mitochondria copy number of young bulls’ spermatozoa compared to their old counterparts during the summer (29 ± 3 vs. 51 ± 6, *p* < 0.001) and winter (27 ± 3 vs. 43 ± 7, *p* < 0.01) seasons. However, sperm-borne mitochondrial protein-coding genes were transcriptionally higher in young bulls throughout the year. Within the same group of bulls, unlike the old bulls, there was a significant (*p* < 0.05) induction in the transcription activity accompanied by a significant (*p* < 0.05) reduction in the mitochondrial copy numbers in the summer (29 ± 3) and winter (27 ± 3) compared to the spring (42 ± 9) and autumn (36 ± 5) seasons in young bulls. Additionally, the pathway enrichment of the top six expressed genes differed between age groups and seasons. In conclusion, under the same quality of semen, the early stages of age are associated with mitochondrial biogenesis and transcription activity dysregulation in a season-dependent manner.

## 1. Introduction

Fertility, which generally refers to the ability to conceive offspring, is a complex and multifactorial trait that greatly influences the livestock industry [1]. The wide use of artificial insemination technology where the semen of one bull can be used to inseminate multitudinous cows makes bull fertility critical in cattle breeding [2]. Bull subfertility includes poor semen quantity and quality significantly attributable to reproductive failures [3]. Several factors such as genetics [4,5], age [4,6], nutrition [7], body condition score [8], management [4], and environmental factors [4,6,9] are found to have influences on semen quality. Conception rate, service per conception, and non-return rate are used to evaluate bull reproductive efficiency; however, the variable conception rate is still determined for bulls with good semen quality [10]. Thus, the so-far used semen quality evaluation parameters still do not fully and accurately estimate male fertility [11]. The common parameters used in semen quality evaluation include sperm concentration, motility, viability, velocity, membrane integrity, acrosome integrity, DNA integrity, and structure abnormalities [12,13,14,15]. Bull fertility estimation based on semen quality evaluation with sensitive approaches such as mRNA profiles is needed [16]. The integration of transcription profiles and dynamics is associated with a better understanding of sperm function and quality to predict potential bull fertility [11,17].

Mitochondria are well-known as central cellular organelles that maintain cellular functions and homeostasis in all cell types. They are mainly involved in energy production but also play vital roles in signal transduction, cell differentiation, apoptosis, and cell cycle control and growth [18,19,20,21]. There is an increasing interest in sperm physiology studies for the implication of the mitochondria as sperm fertility biomarker [22,23,24,25]. Sperm mitochondrial malfunction was reported to be a main contributor in many cases of idiopathic male infertility [26]. Although energy production as the main function of the sperm mitochondria determines sperm motility as a male fertility indicator, sperm mitochondria are also crucial for various sperm characteristics including capacitation, acrosome reaction, hyperactivation, acrosin activity, and DNA integrity [27,28]. Mammalian sperm has a range of 50–75 mitochondria with one copy of mitochondrial DNA (mtDNA) in each [29]. According to genetic databases (NCBI and Ensembl), bovine mtDNA is a double-stranded circular molecule of approximately 16.5 kilobases that exists independently of the nuclear genome and codes for 13 protein-coding genes, 22 tRNAs, and 2 rRNAs.

So far, it is known that the transcription process remains inactive in the mature sperm head for the nuclear DNA; however, the central dogma including transcription and translation processes remains active for the sperm mtDNA. Mature sperm carries different types of RNAs that were found to be released into the oocyte and play a vital role in embryo development [30,31,32,33,34,35,36]. Numerous studies demonstrated the correlation between sperm-borne RNA and fertility, conception rate, and pregnancy success in bovine [11,17,30,37,38,39,40,41]. Previous studies reported the potential translation of the sperm-borne mRNA by the mitochondrial ribosomes, and the translated proteins have roles in sperm capacitation, motility, and pre and post-fertilization processes [30,31,42,43,44]. The mtDNA activity and copy number were found to be influenced by several factors including genetics, age, stress, nutrition, management, cryopreservation, and the quality of the sperm itself [45,46,47]. For instance, mutations in mtDNA genes, namely, *CYTB* and *ATP6*, have been shown to have a major influence on motility, quality, and fertilization capacity [26,48,49]. Furthermore, higher levels of reactive oxygen species were found in spermatozoa with compromised mitochondria and insufficient ATP production. Additionally, there is a correlation between age and the mtDNA copy number with an increased copy number in the sperm cells of old males [50]. The mtDNA copy number may be marked as a sensitive biomarker for semen quality and mitochondrial dysfunction under stress conditions [51,52,53,54]. The sperm mtDNA copy number does not indicate successful fertilization [55]; however, spermatozoa of low-quality semen showed a significant increase in the mtDNA copy number and a decrease in mtDNA integrity [56]. mtDNA content is important for normal sperm function and may help identify markers of male fertility [57].

Moreover, there is considerable interest in genomic selection to identify the desired bull at young ages [58,59]. Moreover, it is highly demanded to collect semen as early as possible from the selected bulls. However, the standard semen quality parameters for young bulls’ semen are not as good compared to the older ages [6,60,61]. Moreover, at the transcriptional level, unlike the young bulls, the old bulls’ spermatozoa showed higher RNA levels of sperm-borne antioxidant transcripts associated with high antioxidant capacity [62]. Although there have been intensive studies on semen quality at phenotypic and molecular levels, limited data about the criteria of accepted semen from young bulls have been reported. Moreover, the potential influence of age and season on the mtDNA transcription activity of young and old bulls’ spermatozoa has not been reported. Therefore, to address these points, this study was conducted on semen samples collected from young and old Holstein bulls during the four seasons of the year and subjected to sperm-borne RNA quantification of the thirteen bovine mitochondrial protein-coding genes. The influence of age and season on the mtDNA copy number and its correlation with the mtDNA transcription activity was also investigated.

## 2. Results

### 2.1. Variation in mtDNA Copy Number Between Young and Old Bulls’ Spermatozoa

**Figure 1:**
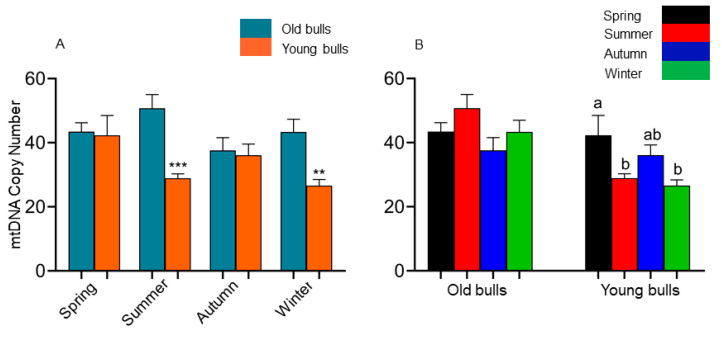
Differential analysis of sperm-borne mitochondrial copy number between the old and young bulls’ spermatozoa (**A**) and between seasons among each group of bulls (**B**). Values are presented as geometric mean ± geometric standard deviation. ** Refers to significant differences, *p* < 0.01; *** refers to significant differences, *p* < 0.001. Different letters refer to significant differences, *p* < 0.05.

A quantitative analysis of the spermatozoa mtDNA copy number was conducted between the spermatozoa collected from young and old bulls throughout the year. For this purpose, the mean copy number derived from the analysis of two mtDNA genes (*ND2* and *CYTB*) was determined. First, we looked at the pattern of the mtDNA copy number in the sperm cells between different ages of bulls, which was significantly reduced in the summer (29 ± 3 vs. 51 ± 6, *p* < 0.001) and winter (27 ± 3 vs. 43 ± 7, *p* < 0.01) spermatozoa of young bulls compared to their old counterparts (Figure 1A). Moreover, group-wise analysis (Figure 1B) revealed that the summer season induced the mtDNA copy number of old bulls’ spermatozoa but significantly reduced it in the young bulls’ counterparts. Moreover, there was a significant reduction in the mtDNA copy number of the young bulls’ spermatozoa during autumn compared with the spring season (Figure 1B).

### 2.2. Differential mRNA Expression Patterns of Mitochondrial Protein-Coding Genes in Old and Young Bulls’ Spermatozoa

**Figure 2:**
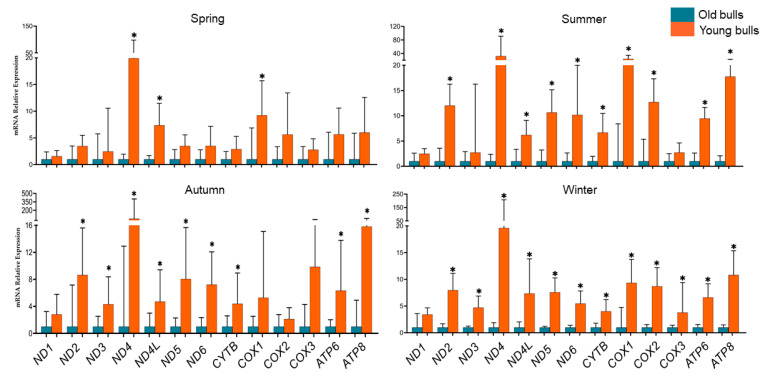
Differential expression analysis of sperm-borne mitochondrial protein-coding genes along the different seasons of the year in the spermatozoa of old and young bulls. Values are presented as geometric mean ± geometric standard deviation. * Refers to significant differences, *p* < 0.05.

To study the consequences of the age of the bull during semen collection on mitochondrial transcription activity during all seasons, we quantified the expression profile of all protein-coding mtDNA genes (13 genes). Therefore, the expression levels between comparable semen samples collected at the same time point from young (*n* = 4, aged 18–24 months old) and old (*n* = 4, aged 40–54 months old) were analyzed. The results presented in Figure 2, indicated that the transcription patterns of mitochondrial genes were higher in young bulls’ spermatozoa throughout the year. All analyzed mitochondrial genes except the *ND1* gene exhibited significantly high levels in young bulls’ spermatozoa during winter (Figure 2). Furthermore, young bulls’ spermatozoa showed significantly high mRNA levels of all genes except for the *ND1*, *ND3*, and *COX3* genes during the summer season and the *ND1*, *COX1*, *COX2*, and *COX3* genes during the autumn season (Figure 2). However, during the spring season, only the *ND4L* and *COX1* genes showed significantly high sperm-borne mRNA levels of young bulls’ spermatozoa (Figure 2). The *ND1* gene exhibited non-significant high levels in young bulls’ spermatozoa compared to old bull counterparts throughout the year (Figure 2). Furthermore, data presented in Table 1, for the co-expression score for the significant genes throughout the year, showed that *ND4* and *ND5* genes had the highest co-expression score, while a moderate co-expression score was pointed for the *ATP8* and *COX1* genes.

### 2.3. Comparative Expression Analysis of All Mitochondrial Protein-Coding Genes Among Each Age Group of Bulls Throughout the Year

**Figure 3:**
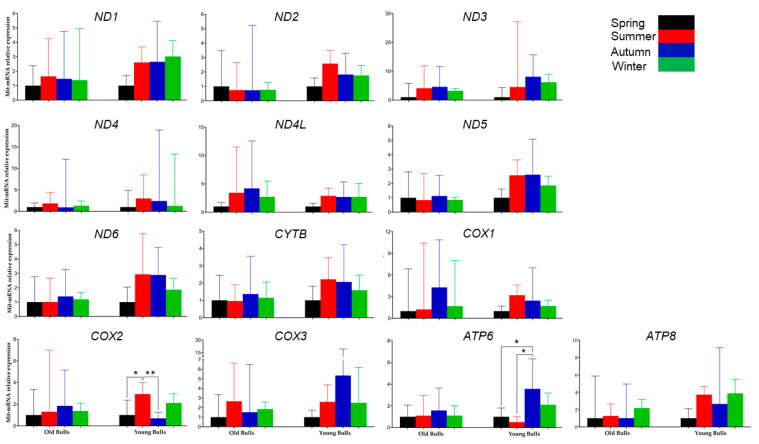
Differential expression analysis of sperm-borne mitochondrial protein-coding genes between the seasons of the spermatozoa of old and young bulls. Values are presented as geometric mean ± geometric standard deviation. * Refers to significant differences, *p* < 0.05; ** refers to significant differences, *p* < 0.01.

To identify whether the mitochondria transcriptional level of each protein-coding gene was influenced by the season, the mRNA expression was analyzed by comparing the differences between the four seasons within each group of young and old bulls separately. The results revealed that the season had no significant influence on the transcription level of each mitochondrial gene in spermatozoa collected from old bulls. However, the young bulls during the spring showed low sperm-borne RNA levels of all mitochondrial genes compared to the other three seasons (Figure 3). Moreover, the young bulls’ spermatozoa during summer compared to spring and autumn showed significantly higher levels of the *COX2* gene. However, during autumn, the mRNA level of sperm-borne *ATP6* was highly significant compared to spring and summer in young bulls’ spermatozoa (Figure 3).

### 2.4. Differential Abundance of the Mitochondrial Expressed Genes in Old and Young Bulls Throughout the Year

**Figure 4:**
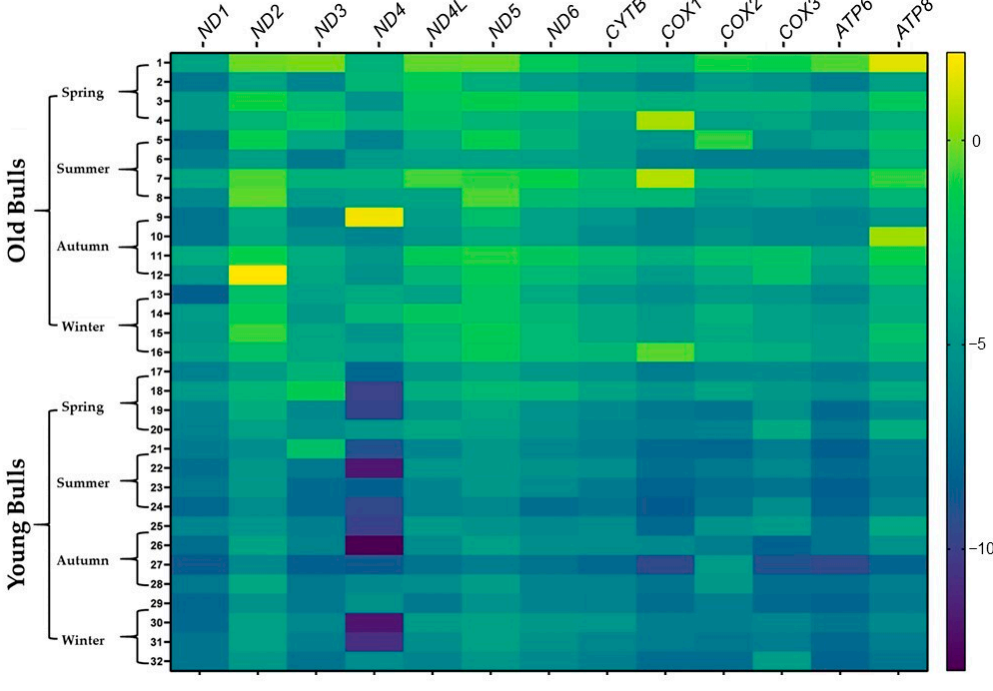
The heatmap displays the delta Ct values of the protein-coding mitochondrial genes in old and young bulls throughout the year. Variations in delta Ct are represented by color gradation within the range from −10 to 0; bright color refers to a high delta Ct value that reflects low expression, and dark color refers to a low delta Ct value that reflects higher expression.

**Figure 5:**
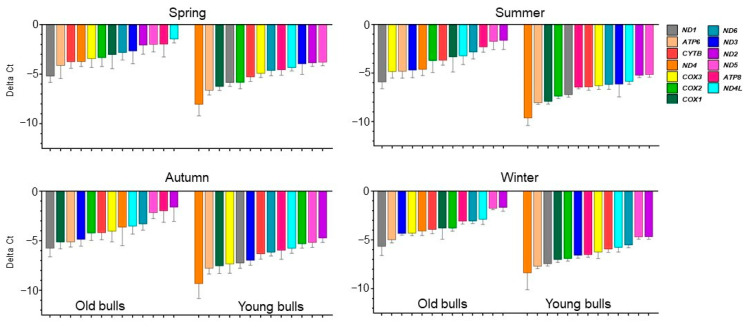
The abundance of the spermatozoa mitochondrial protein-coding genes throughout the year. Values are presented as mean ± standard error mean.

To illustrate the abundance of the genes in each bull group during the same season the analyzed genes were sorted according to their delta Ct (cycle threshold) values. The analysis demonstrated that the abundance of the genes differed between old and young bulls within the same season; moreover, it differed between seasons within the same bull group (Figure 4 and Figure 5). The mRNA of the *ND1* gene was the most abundant among the thirteen genes in spermatozoa of old bulls throughout the year. However, *ND4* and *ATP6* were the most abundant transcripts, while *ND2* and *ND5* were the lowest abundant ones among the sperm-borne analyzed genes in young bulls throughout the year (Figure 4 and Figure 5).

### 2.5. Gene Ontology Analysis of the Top 6 Abundant Protein-Coding Mitochondrial Genes Throughout the Year of Old and Young Bulls’ Spermatozoa

**Figure 6:**
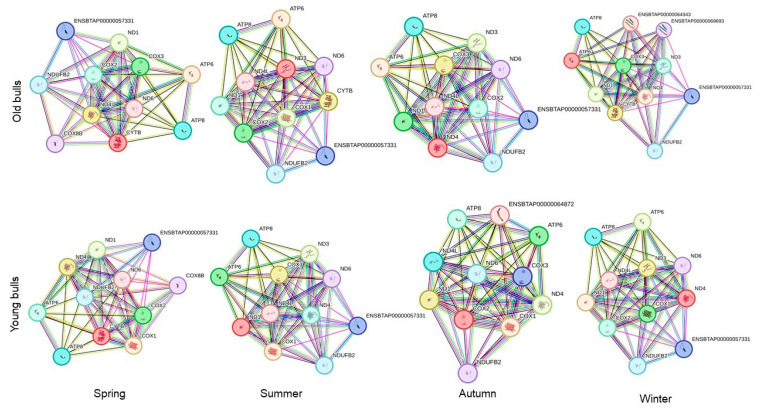
The protein–protein interaction network analyzed by String software version 12.0 for the top six abundant mitochondrial protein-coding genes in each group of bulls throughout the year. An edge was drawn with up to seven differently colored lines that represent the existence of the seven types of evidence used in predicting the associations. The red line represents the fusion evidence; the green line represents the neighborhood evidence; the blue line represents the co-occurrence evidence; the purple line represents the experimental evidence; the yellow line represents the text-mining evidence; the light blue line represents the database evidence; and the black line represents the co-expression evidence.

**Figure 7:**
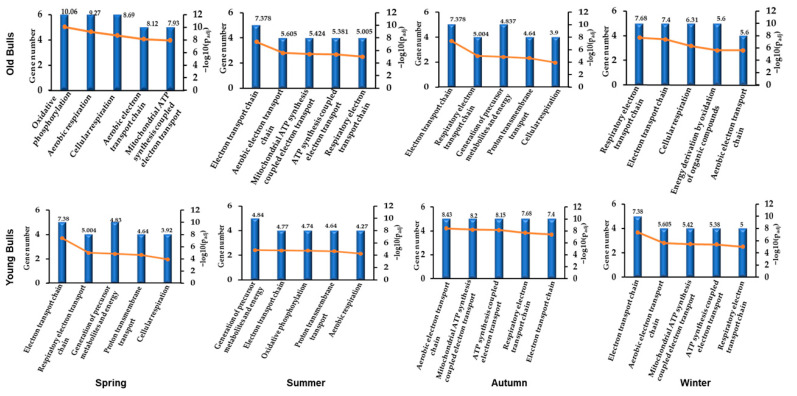
The gene ontology distribution of differential biological pathways according to the top six abundant mitochondrial protein-coding genes in each group of bulls throughout the year. The *X*-axis shows the top five pathways of the mitochondria; the left *Y*-axis shows the number of genes involved in each particular pathway; the right *Y*-axis shows the −log10 of the adjusted *p*-values (p_adj_).

The in silico analysis for the top six abundant expressed genes in each bull group for every season using gene ontology and protein–protein interaction online tools showed eleven protein-to-protein interactions in old and young bulls (Figure 6). The ND1 had the highest number of interactions in old bulls throughout the year. However, in young bulls, the ND4 showed high interactions with the other 10 proteins (Figure 6). Furthermore, the pathway enrichment analysis of these top six abundant genes showed nine signaling pathways including oxidative phosphorylation, aerobic respiration, cellular respiration, aerobic electron transport chain, ATP-synthesis-coupled electron transport, electron transport chain, the generation of precursor metabolites and energy, proton transmembrane transport, and energy derivation by the oxidation of organic compounds that were differentially enriched by their *p*-value for the top five biological pathways between the seasons and age groups (Figure 7).

## 3. Discussion

The increased improvement in genomic selection accompanied by the vast use of cryopreserved semen in artificial insemination and in vitro embryo production applications increased the interest in semen collection from young bulls at early stages [58,59]. It has been reported that semen quality determines male fertility [10,11,63]. In sperm cells, mitochondria are involved in sperm motility, capacitation, acrosome reaction, and other functions that maintain sperm functionality [25,26,64]. They are considered biomarkers for fertility and sperm fertilization ability [22,23,24]. In the same line, the current study aimed to determine the mtDNA copy number in young (*n* = 4, aged 18–24 months old) and old (*n* = 4, aged 40–54 months old) Holstein bulls and its association with the transcription activity of all mitochondrial protein-coding genes. Furthermore, the influence of the season on the spermatozoa mtDNA copy number and expression activity was investigated. The mtDNA copy number has been defined as the number of mtDNA copies per nuclear DNA copy and is correlated to the mitochondrial function [52]. In germ cells, the mitochondrial DNA copies are about 150,000 copies in the oocyte; however, they present with a lower number (100 copies) in sperm cells [65]. The reduction in the mtDNA copy number is vital for sperm growth, maturation, and subsequent sperm functions [65]. In agreement with that, the mtDNA copy number in the present study was within the normal mtDNA copy number range of 50–75 mtDNA for good, motile sperm [29]. However, the young bulls’ spermatozoa in our study showed a lower number than old bulls, and there was a high significant reduction in the mtDNA copy number of young bulls’ spermatozoa during summer and winter than was found in old counterparts (Figure 1A,B). Previous studies demonstrated that the mtDNA copy number is dramatically influenced by age changes [47]. The low mtDNA copy number is associated with mortality [66]. Moreover, the mtDNA copy number is influenced by the stress [66,67]. The results shown in Figure 1 illustrated that the summer season had an influence on increasing the mtDNA copy number in old bulls’ spermatozoa unlike in young ones, which indicates the potential normal functionality of the mitochondria in the supposed old bulls in the current study. The mtDNA copy number directly correlates with energy reserves, oxidative stress, and mitochondrial membrane potentiality [67]. The mtDNA copy number indicates the mitochondrial function, activity, and biogenesis [65]. The reduction in mtDNA copy number was found to be associated with cellular dysfunction [68]. The mtDNA replication is one main base for mitochondrial biogenesis where the replication process relies on nuclear protein-coding genes. Peroxisome-proliferator-activated receptor gamma coactivator 1 alpha (PGC-1D) induces the expression of nuclear erythroid-related factor 2 (*NRF2*), nuclear respiratory factor 1 (*NRF1*), and mitochondrial transcription factor A (*TFAM*) genes, which regulate mitochondria biogenesis [69]. Our previous results showed significantly high sperm-borne RNA content of *NRF2* transcripts in old bulls’ spermatozoa compared to young ones, which could be in agreement with higher mtDNA copy number in old bulls compared with young ones in the present study [62]. It was reported that the change in the mtDNA copy number is correlated with the transcription activity of mitochondrial protein-coding genes [70].

At the transcription level, the mature sperm cell is known to have an absence of transcription and translation processes [36,71]. However, these processes are active for sperm mitochondrial DNA to maintain mitochondrial functions [72]. In the present study, we quantified the sperm-borne mRNA content of all mitochondrial protein-coding genes (13 genes) in young and old bulls’ spermatozoa. All mitochondrial protein-coding genes were detected in spermatozoa of both young and old bulls (Figure 4). However, the results revealed that throughout the year generally, the young bulls’ spermatozoa had higher mRNA levels of all analyzed genes (Figure 2). The data indicate an increase in the transcription activity in young bulls’ sperm-borne mitochondria, which may be a consequence of transcription dysregulation through the loss of epigenetic regulation mechanisms such as DNA methylation. In agreement with this hypothesis, during the summer and winter seasons, there was a significant reduction in mtDNA copy number in young bulls’ spermatozoa (Figure 1) associated with a significant increase in the expression levels of mitochondrial genes (Figure 2). There is evidence indicating that both mitochondrial DNA transcription and replication could be regulated through epigenetic mechanisms [73]. Another study has suggested that DNA methylation as an epigenetic mechanism may have a potential role in regulating the sperm mtDNA copy number [74]. Furthermore, mtDNA methylation may be influenced by environmental factors [75]. In the same study, a negative correlation was found between mtDNA methylation and gene expression level [75]. Our findings revealed that the season had a higher influence on the expression patterns of the studied genes in young bulls than determined in old ones (Figure 3). Although the differences were non-significant, the spring season associated with low mRNA levels of sperm-borne mitochondrial genes (Figure 3). However, a significantly high level of the *COX2* gene was detected in young bulls’ spermatozoa during summer compared to spring and autumn. Additionally, in the same bull group, the sperm-borne mRNA level of the *ATP6* gene was significantly reduced during summer compared to autumn (Figure 3). The mitochondrial cytochrome *c* oxidase (COX) protein family is the primary set of oxygen consumption and is central in ATP synthesis through aerobic energy generation [76]. Previous studies demonstrated the presence of the *COX2* gene in normal male reproductive cell types and spermatozoa; however, higher levels of the gene were determined under pathological conditions and low fertile individuals [77,78]. The *ATP6* gene encodes for the ATP synthase membrane subunit 6 protein, which is an enzyme involved in oxidative phosphorylation, electron transport chain, and proton transmembrane transport biological pathways for normal mitochondrial function [79,80]. Reduction in the expression level of *ATP6* was associated with cellular dysfunction and low maturation in bovine oocytes [81].

The abundance of a particular gene within a gene cluster indicates the alteration of a specific pathway or biological process [82]. For instance, the mRNA transcript of the nuclear gene *YWHAZ* was one of the most abundant genes in bovine spermatozoa [83] as it has a role in regulating spermatogenesis and acrosomal reactions [84]. Furthermore, the mRNA along with the protein of the mitochondrial COX1 gene was abundant in bovine spermatozoa [83], and the alteration of the mitochondrial genes results in mitochondrial dysfunction and impaired fertility [85]. In the current study, all mitochondrial protein-coding genes under investigation were detected in RNA isolated from the spermatozoa of both young and old bulls (Figure 4). However, the abundance of the mRNA of these genes differed between bull groups and seasons. As shown in Figure 4, the delta Ct values were low (high abundant) for a particular gene in one group and high (low abundant) in another group. Accordingly, we sorted the genes by their Ct values (Figure 5). Throughout the year, the mRNA of the *ND1* gene was the topmost abundant compared to other genes in old bulls’ spermatozoa. Complex I (NADH: ubiquinone oxidoreductase) is a mitochondrial enzyme complex responsible for proton generation and electron transport in the mitochondrial inner membrane essential for oxidative phosphorylation and ATP production [86]. The dysregulation of NAD1 protein was found to adversely influence complex I activity, resulting in low energy production and subsequently sperm motility [87]. However, the *ND4* and *ATP6* genes were the topmost abundant throughout the year while the *ND2* and *ND5* were the least abundant compared to other genes in young bulls’ spermatozoa (Figure 5). All studies demonstrated the impact of mitochondrial expression gene loss; however, suggestions regarding higher expression are limited. Therefore, in the current study, the mRNA analysis together with the mtDNA copy number can suggest that the imbalance between mitochondrial genes may result in mitochondrial dysfunction and the loss of sperm function. Moreover, the season influenced the differential abundance between the mitochondrial genes (Figure 5) particularly in old bulls’ spermatozoa, indicating the potential use of the mitochondrial transcript abundance as a biomarker for sperm quality. Furthermore, an in silico analysis was performed for the protein–protein interaction and pathway enrichment for the top six abundant genes in each group of bulls during every season. The results showed eleven protein-to-protein interactions where the ND1 for the old bulls and ND4 for the young bulls showed high interactions with the other 10 proteins (Figure 6). Moreover, these top six abundant genes in each group showed the involvement of the same pathways linked with mitochondrial function and energy metabolism differentiated by age and season (Figure 7). The current study was limited by the number of animals and relied on −80 °C frozen semen. Therefore, further analyses using more bulls and sperm phenotypes on fresh and cryopreserved semen could help to identify particular balanced mitochondrial transcription abundance as a biomarker for sperm quality within young bulls.

In conclusion, our study was the first to investigate the association between the mtDNA copy number and the transcription activity of all mitochondrial protein-coding genes in young and old bulls’ spermatozoa throughout the year. There was a significant negative influence of summer and winter seasons on young bulls’ sperm-borne mitochondrial regulation, which resulted in a low copy number and the transcription dysregulation of all mitochondrial protein-coding genes and the subsequent biological pathways enrichment. Further investigations are ongoing on the mtDNA epigenetic regulatory mechanisms associated with mtDNA replication and transcription activity.

## 4. Materials and Methods

### 4.1. Experimental Design

The study investigations were performed on Black Holstein semen samples collected and cryopreserved at the RSH (Rinderzucht Schleswig-Holstein, Neumünster, Germany) station. The semen was collected from eight healthy bulls including young (*n* = 4, aged 18–24 months old) and old (*n* = 4, aged 40–54 months old) bulls during winter (January), spring (April), summer (August), and autumn (November) seasons using artificial vagina protocol. All morphological parameters for sperm quality before cryopreservation were examined at the RSH station. All selected samples were pointed with good semen quality parameters of more than 80%. The semen samples were cryopreserved using the protocol by the RSH station and stored in liquid nitrogen. Thereafter, straws were transferred to the lab and kept at −80 °C until use. The straws were thawed in the lab, and the semen samples were washed twice in PBS (phosphate buffer saline). The washed spermatozoa were subjected to the sperm-borne DNA and RNA extraction. After that, the mitochondrial DNA copy number analysis was performed using the extracted DNA and qPCR (quantitative polymerase chain reaction). In parallel, the RNA was subjected to cDNA synthesis and mRNA expression analysis of the sperm-borne 13 mitochondrial protein-coding genes. According to the results, the genes were sorted according to their delta Ct values. Then the top six abundant genes were used for protein–protein interaction and pathway enrichment analyses using gene ontology online tools.

### 4.2. DNA Extraction and Quality Control

The DNA from sperm cells (one straw per animal) was extracted using a manual protocol. Briefly, the straws were thawed at 37 °C and diluted with PBS then centrifuged at 13,000 rpm for 10 s. Thereafter, the pellets were washed twice with PBS before resuspension in with a lysis buffer containing 100 mM NaCl, 10 mM Tris-base pH 8.2, 2 mM EDTA pH 8.2, 0.5 M DTT, 10% SDS, and proteinase K. The samples suspended in the lysis buffer were incubated overnight at 65 °C. The lysate was then mixed with 6 M NaCl (*V*/*V*) and centrifugated for 13 min at 13,000 rpm. The clear supernatant was transferred into a new tube and mixed with 100% ethanol and centrifuged for 1 min at 13,000 rpm. Finally, the supernatant was discarded, and the pellets were dissolved in 37 °C Tris EDTA buffer. The DNA was then subjected to quality and quantity assays. For that, the DNA concentration and purity were performed using a NanoDrop 1000 Spectrophotometer (PEQLAB Biotechnologie GmbH, Erlangen, Germany). Furthermore, the integrity of the DNA was performed by loading 100 ng from each DNA sample on 1% agarose gel run at 80 voltages for 1 h. Finally, the pictures were developed using a gel documentation device (Bio-Rad Laboratories, Inc., Hercules, CA, USA).

### 4.3. RNA Extraction and cDNA Synthesis

The sperm-borne total RNA was extracted using the RNeasy Mini Kit (Qiagen, Hilden, Germany) according to the manufacturer’s instructions with some modifications. Briefly, the straws (two straws per animal) were thawed at 37 °C and diluted with PBS then centrifuged at 13,000 rpm for 10 s. The pellets were washed twice with PBS and incubated with Qiazol buffer with beta-mercaptoethanol for 15 min followed by the kit manufacturer’s instructions. After the washing steps, the columns were subjected to DNase I treatment and incubated for 15 min to remove residual DNA contamination. Subsequently, after the washing steps, the RNA was eluted in 50 µL elution buffer. The RNA concentration and purity were determined using a NanoDrop 1000 Spectrophotometer (PEQLAB Biotechnologie GmbH, Erlangen, Germany). Afterward, the RNA samples were standardized for 120 ng using RNAse-free water for cDNA synthesis. The cDNA was synthesized by reverse transcription using the first strand cDNA synthesis kit (Thermofisher Scientific, Dreieich, Germany), following the manufacturer’s instructions.

### 4.4. Mitochondrial DNA Copy Number

The mitochondrial DNA (mtDNA) copy number of old and young bulls’ spermatozoa was identified by quantification analysis using the extracted DNA. The amplification of DNA was performed via qPCR and SsoAdvanced Universal SYBR^®^ Green Supermix (Bio-Rad Laboratories GmbH, Feldkirchen, Germany) using primers (Table 2) designed for the nuclear (n) (*GAPDH*) and mitochondrial (mt) (*ND2* and *CYTB*) genes. For that, serial concentrations (10, 30, 50, and 70 ng) of DNA were used in a total amplification volume of 20 µL for each gene. The relative mtDNA copy number was calculated using the following equation: mtDNA copy number = 2^1+(Ctn_gene-Ctmt_gene)^ [88].

### 4.5. Sperm-Borne mRNA Expression Analysis

The sperm-borne mRNA levels of all the thirteen protein-coding mitochondrial genes named *ND1*, *ND2*, *ND3*, *ND4*, *ND4L*, *ND5*, *ND6*, *CYTB*, *COX1*, *COX2*, *COX3*, *ATP6*, and *ATP8* were determined using primers listed in Table 2, designed for each gene using the Primer3 online tool version 4.1.0. The amplification was performed by qPCR using the synthesized cDNA and SsoAdvanced Universal SYBR Green Supermix (Bio-Rad Laboratories GmbH, Feldkirchen, Germany). A total reaction of 20 µL was run using the following program: 95 °C for 3 min (1 cycle), followed by incubation at 95 °C for 15 s, and then at 60 °C for 45 s (40 cycles). Finally, a melting curve was performed. The relative mRNA expression analysis was performed using the delta–delta Ct (2^−△△CT^) method and the *GAPDH* and *B2M* genes as endogenous normalizers.

### 4.6. Gene Ontology Enrichment and Protein–Protein Interaction Analysis Network

The gene enrichment and protein–protein interaction between the sperm-borne abundant mitochondrial protein-coding genes were analyzed using g:Profiler (https://biit.cs.ut.ee/gprofiler/gost, accessed on 1 October 2024) and STRING 12.0 (https://string-db.org, accessed on 26 September 2024) online tools. All the pathway enrichment and protein–protein interactions were created using text mining, experiments, databases, co-expression, neighborhood, gene fusion, and co-occurrence with a high confidence level.

### 4.7. Statistical Analysis

The mRNA expression and mtDNA copy number data were represented using the geometric mean ± geometric standard deviation. Statistical analyses were performed using the GraphPad Prism10 software. The statistical differences between the four different seasons within the same age group of bulls were analyzed using one-way ANOVA followed by a multi-comparison Tukey test. However, statistical differences between young and old bulls within each season were analyzed using a student t-test (unpaired and two-tailed). Statistical significance was considered at *p* < 0.05.

## Figures and Tables

**Table 1:** *Bos taurus* mitochondrial protein-coding genes, displayed from highest to lowest values of co-expression score.

Node1	Node2	Score	Node1	Node2	Score	Node1	Node2	Score
*ND5*	*ND4*	0.989	*COX3*	*COX1*	0.942	*ND6*	*COX3*	0.322
*ND4*	*CYTB*	0.989	*COX1*	*COX3*	0.942	*COX3*	*ND6*	0.322
*ND4*	*ND2*	0.989	*ND4*	*COX1*	0.940	*ND1*	*ATP8*	0.311
*ND4*	*ND5*	0.989	*ND3*	*ND1*	0.940	*ATP8*	*ND1*	0.311
*ND2*	*ND4*	0.989	*ND1*	*ND3*	0.940	*ND5*	*ATP8*	0.302
*CYTB*	*ND4*	0.989	*COX2*	*COX1*	0.940	*ATP8*	*ND5*	0.302
*ND5*	*ND2*	0.985	*COX1*	*ND4*	0.940	*CYTB*	*ATP8*	0.285
*ND2*	*ND5*	0.985	*COX1*	*COX2*	0.940	*ATP8*	*CYTB*	0.285
*CYTB*	*COX3*	0.985	*CYTB*	*COX1*	0.938	*COX2*	*ATP8*	0.273
*COX3*	*CYTB*	0.985	*COX1*	*CYTB*	0.938	*ATP8*	*COX2*	0.273
*ND5*	*CYTB*	0.984	*ND5*	*ATP6*	0.936	*ND6*	*COX1*	0.212
*ND4*	*COX3*	0.984	*ATP6*	*ND5*	0.936	*COX1*	*ND6*	0.212
*ND2*	*CYTB*	0.984	*ND3*	*ND2*	0.914	*COX3*	*ATP8*	0.205
*CYTB*	*ND5*	0.984	*ND2*	*ND3*	0.914	*ATP8*	*COX3*	0.205
*CYTB*	*ND2*	0.984	*ND6*	*ND5*	0.910	*COX1*	*ATP8*	0.123
*COX3*	*ND4*	0.984	*ND5*	*ND6*	0.910	*ATP8*	*COX1*	0.123
*ND4*	*ATP6*	0.979	*ND4L*	*ND4*	0.891	*ND2*	*COX3*	0.109
*ND2*	*ND1*	0.979	*ND4*	*ND4L*	0.891	*COX3*	*ND2*	0.109
*ND1*	*ND2*	0.979	*ND5*	*ND3*	0.884	*ND2*	*ND3*	0.103
*ATP6*	*ND4*	0.979	*ND3*	*ND5*	0.884	*ND3*	*ND2*	0.103

**Table 2:** List of forward (F) and reverse (R) primers used for the mt-DNA copy number and the sperm-borne mRNA analyses.

Symbol	Gene Name	Primer Sequence (5′-3′)	Size (bp)
*ND1*	NADH Oxidoreductase Core Subunit 1	F: 5′CACTACGACCCGCTACATCT3′ R: 5′AGTTGGAAGCTCAGCCTGAT3′	195
*ND2*	NADH Oxidoreductase Core Subunit 2	F: 5′ATCACAACCCACGAGCTACA3′ R: 5′GATGCCCTGTGTTACTTCTGG3′	227
*ND3*	NADH Oxidoreductase Core Subunit 3	F: 5′ATCGCATTCTGACTTCCCCA3′ R: 5′CAGTGGTAGGAGGAGTGCAA3′	168
*ND4*	NADH Oxidoreductase Core Subunit 4	F: 5′GGAAACCAAACAGAACGCCT3′ R: 5′AGGTAGTCAAAGGTGGAGGC3′	243
*ND4L*	NADH Oxidoreductase Core Subunit 4L	F: 5′AGCAGCCCTAACAATCCTCA3′ R: 5′AGCATTGGAGTAAGTTGAGGTT3′	167
*ND5*	NADH Oxidoreductase Core Subunit 5	F: 5′TGAGAAGGCGTCGGAATCAT3′ R: 5′GGATTTTCCGGTTGCAGCTA3′	243
*ND6*	NADH Oxidoreductase Core Subunit 6	F: 5′ACTGGCTTGTTGATGGAGTTC3′ R: 5′TAAAGCCGCAATCCCTATGG3′	156
*CYTB*	Cytochrome B	F: 5′TACCCATATCTGCCGAGACG3′ R: 5′TGGTGATGACTGTTGCTCCT3′	245
*COX1*	Cytochrome C Oxidase Subunits I	F: 5′AGGAGCCATCAACTTCATTACA3′ R: 5′AGGTTCCGGTCTGTTAATAGCA3′	168
*COX2*	Cytochrome C Oxidase Subunits II	F: 5′CCAGGGGAGCTACGACTATT3′ R: 5′GACCCGCAAATTTCTGAGCA3′	218
*COX3*	Cytochrome C Oxidase Subunits II	F: 5′ATCCGAGAAAGCACCTTCCA3′ R: 5′TGTTGAGCAGTGGGACTTCT3′	217
*ATP6*	ATP Synthase Membrane Subunits 6	F: 5′ACCCACTCCACTAATCCCAATA3′ R: 5′GCAAGTGTAGCTCCTCCGAT3′	141
*ATP8*	ATP Synthase Membrane Subunits 6	F: 5′CCGCAACTAGACACGTCAAC3′ R: 5′TGTTTCTCAAGGGGTGTTTTGT3′	156
*GAPDH*	Glyceraldehyde-3-phosphate dehydrogenase	F: 5′CCCAGAATATCATCCCTGCT3′ R: 5′CTGCTTCACCACCTTCTTGA3′	369
*B2M*	Beta-2-microglobulin	F: 5′TCCAGCGTCCTCCAAAGATT3′ R: 5′CCTTGCTGTTGGGAGTGAAC3′	222

## Data Availability

Data are contained within the research article.
